# Combining Activation‐Induced Markers With PD‐L1 Selectively Enhances Detection of Antigen‐Specific T Cells in Virus‐Infected Individuals

**DOI:** 10.1155/jimr/1284907

**Published:** 2026-02-04

**Authors:** Christine Kreher, Laura Y. L. Kummer, Maryse Tempert, Veronique A. L. Konijn, Gertjan Wolbink, Filip Eftimov, Taco W. Kuijpers, Theo Rispens, S. Marieke van Ham, Anja ten Brinke

**Affiliations:** ^1^ Sanquin Research and Landsteiner Laboratory, Amsterdam UMC, University of Amsterdam, Amsterdam, Netherlands, uva.nl; ^2^ Department of Neurology and Neurophysiology, Amsterdam UMC, Location AMC, Amsterdam, Netherlands, amc.nl; ^3^ Amsterdam Rheumatology and Immunology Center, Amsterdam UMC, Location Reade, Amsterdam, Netherlands, amc.nl; ^4^ Department of Pediatric Immunology, Rheumatology and Infectious Disease, Amsterdam UMC, Amsterdam, Netherlands, amc.nl; ^5^ Swammerdam Institute for Life Sciences, University of Amsterdam, Amsterdam, Netherlands, uva.nl

**Keywords:** AIM, Boolean gating, CD4, CD8, CMV, SARS-CoV-2, T helper cells, vaccination

## Abstract

Detection and characterization of antigen‐specific T cells are important for studying immune responses upon infection, vaccination, or autoreactivity. The activation‐induced marker (AIM) assay is a robust technique to identify and characterize antigen‐specific CD4 and CD8 T cells. However, there is variability in the AIM assay, particularly in the type and number of activation markers used. In this study, we set out to define which marker combinations are most suited to optimally detect antigen‐specific CD4 and CD8 T cells and if certain marker combinations preferentially detect specific CD4 T helper subsets. A multiparameter flow cytometry panel, including six common activation markers: CD40L, CD137, CD69, OX40, CD25, and PD‐L1, was used for detecting antigen‐specific T cells following infection (SARS‐CoV‐2 and CMV) or vaccination (mRNA‐1273 SARS‐CoV‐2). We demonstrate that combining multiple activation markers increases the detection frequency of antigen‐specific CD4 T cells compared to commonly used dual marker combinations. In addition, marker combinations including PD‐L1 detected a higher frequency of antigen‐specific CD4 T cells in SARS‐CoV‐2 and CMV infected but not in SARS‐CoV‐2–vaccinated individuals. Certain dual marker combinations preferentially detected specific CD4 T helper subsets. The majority of antigen‐specific CD8 T cells were captured by the dual combination of CD69 plus CD25. In conclusion, combining CD137, CD69, OX40, CD25, and PD‐L1 in an AIM assay results in robust and optimal detection of both specific CD4 T helper subsets and CD8 T cells in different antigenic contexts.

## 1. Introduction

T cells are a major constituent of the adaptive immune response in both infectious and noninfectious diseases. In order to study T cell responses to a pathogen, autoantigen, or vaccine, antigen‐specific T cells need to be identified and characterized [1, 2]. The identification of antigen‐specific CD4 T cells is particularly challenging, as they are often present at low frequency in circulation. Once identified, characterizing their specific T helper profile can be relevant.

Currently, different platforms are available to detect antigen‐specific T cells. The activation‐induced marker (AIM) assay is a frequently used method. The major advantage of this assay compared to other approaches is that both antigen‐specific CD4 and CD8 T cells can be detected and characterized simultaneously [1, 3, 4]. In this assay, whole blood or PBMCs are most often used as starting material, allowing autologous antigen presentation. During incubation with either whole protein antigens or peptide pools, antigen‐presenting cells (APCs) process them and present peptide epitopes via their MHC class I or MHC class II molecules. Upon engagement with their cognate peptide–MHC complex, antigen‐specific T cells upregulate expression of AIMs on the cell surface within 6–48 h of the start of in vitro culture, and these markers are measured in the AIM assay [1].

Preferably, MHC class I or II multimers are used to quantify and phenotypically characterize antigen‐specific T cells directly ex vivo, thereby circumventing the need for in vitro culture [5, 6]. The major downside of this technique is the requirement for knowledge on the HLA types and the antigenic peptides presented by these HLA molecules. The information is often not available, especially not for CD4 T cells, limiting broad application. Other platforms, such as proliferation assays, capture the proliferative response of T cells upon antigenic challenge [7, 8]. However, proliferation assays require long‐term in vitro culture, which affects the initial frequency and phenotype of antigen‐specific T cells and potentially underestimates the frequency of hypoproliferative cells like Treg cells [9]. Assays like ELISpot/FluoroSpot, which are based on cytokine expression of T cells following antigenic stimulation [10], are limited in detecting the whole breadth of the antigen‐specific T cell response, as only a limited number of cytokines can be detected at once [11]. Therefore, the AIM assay is the preferred method for detecting the broad spectrum of antigen‐specific T cells.

Despite its common application, the AIM assay is not standardized, and a variety of AIM assays are used by different research groups. This complicates the reproducibility and comparison of data between different studies. The activation markers CD25 (IL‐2R*α*), CD40L (CD154), CD137 (4‐1BB), and CD69 are the most commonly used cell surface receptors for detecting antigen‐specific CD4 or CD8 T cells [1, 12]. Moreover, OX40 and PD‐L1 have been described more recently for their application in the AIM assay [4, 13, 14]. Often, a dual marker combination is used [1, 14, 15] to avoid high nonspecific signal (background). This background is either caused by constitutive expression of activation markers on certain T cell subsets, as in the case of CD25 expression on regulatory T cells [16], or by TCR–independent induction of the markers, as is the case with CD69 [17–19]. The dual marker combinations OX40 plus CD137 and CD69 plus CD137 were most commonly used to analyze SARS‐CoV‐2–specific CD4 and CD8 T cells, respectively, in the recent COVID‐19 pandemic [12]. However, it remains unclear whether all antigen‐specific CD4 and CD8 T cells upregulate the same activation markers upon TCR stimulation and if different CD4 T helper subsets preferentially express certain markers. Consequently, more than two activation markers might be required to capture the whole spectrum of the antigen‐specific T cell response. This highlights the need to further characterize the AIM assay by studying how different marker combinations affect the frequency and phenotype of the detected antigen‐specific T cells.

In this study, a multiparameter flow cytometry panel including six previously described activation markers [1]: CD40L, CD137, CD69, OX40, CD25, and PD‐L1, was used to quantify and characterize antigen‐specific CD4 and CD8 T cells against severe acute respiratory syndrome coronavirus 2 (SARS‐CoV‐2) spike or cytomegalovirus (CMV) pp65 following viral infection (SARS‐CoV‐2 and CMV) or mRNA vaccination (SARS‐CoV‐2). We demonstrate that the combination of two markers does not capture the whole breadth of the spike– or CMV–specific CD4 and CD8 T cell response. By combining CD137, CD69, OX40, CD25, and PD‐L1, we could enhance the detection frequency of antigen‐specific CD4 T cells. The dual combination of CD69 plus CD25 was sufficient for the detection of antigen‐specific CD8 T cells. Furthermore, our analysis revealed that the use of dual marker combinations can skew T cell detection towards certain T helper populations. In conclusion, we demonstrate that AIM assays based on one dual marker combination may underestimate the quantity of the antigen‐specific T cell response and introduce bias in the detected CD4 T helper phenotype. By combining multiple activation markers, the AIM assay can more effectively detect low‐frequency antigen‐specific T cells across different sample types.

## 2. Methods

### 2.1. Study Participants and Sample Collection

Peripheral blood mononuclear cells (PBMCs) were obtained in 2020 from healthy COVID‐19 convalescent blood donors at the Sanquin Blood Supply Foundation of the Netherlands. In addition, PBMCs were collected from healthy SARS‐CoV‐2–vaccinated donors participating in a Dutch national cohort study on SARS‐CoV‐2 vaccination [20]. Study protocols were approved by the ethics advisory council of Sanquin Blood Supply Foundation and the medical ethical committee (NL74974.018.20 and EudraCT 2021‐001102‐30), respectively, for the two study cohorts. All participants provided written informed consent. PBMCs were stored in liquid nitrogen until use.

Healthy SARS‐CoV‐2–vaccinated donors received two doses of mRNA‐1273 (Moderna) at a 6‐week interval. PBMCs were collected 7–8 days (median: 7, T7d) and 6.2–6.6 months (median: 6.2, T6m) after the second vaccination (Supporting Information 1: Table S1). Vaccinated donors were tested for previous COVID‐19 infection based on nucleocapsid (N) antibodies at the time of vaccination. The COVID‐19 convalescent cohort consisted of healthy blood donor volunteers who were sampled 30–59 days (median: 54.4, T55d) and 6.5–8.2 months (median: 7.3, T7m) after de novo SARS‐CoV‐2 infection in 2020 (Supporting Information 1: Table S1). SARS‐CoV‐2 vaccinations were not available at that time yet. COVID‐19 infection was confirmed via anti‐RBD, anti‐spike, and/or anti‐N serology, and date of COVID‐19 symptom onset was obtained via a questionnaire. CMV status of convalescent donors was determined using anti‐CMV IgG detection.

### 2.2. AIM Assay

PBMCs were thawed in Iscove’s Modified Dulbecco’s Medium (IMDM, ThermoFisher) with 5% fetal calve serum (FCS), 1% penicillin/streptavidin, and 0.1% DNAse and centrifuged and resuspended in IMDM with 5% FCS, 5% human serum (HS), and 1% penicillin/streptavidin. PBMCs were plated at 2 × 10^6^ cells per well in 96‐flat bottom well plate and stimulated with a spike or CMV pp65 peptide pool (15‐mer with 11aa overlap, Miltenyi Biotec; at 0.6 nmol of each peptide/mL) for 18 or 24 h at 37 °C. An unstimulated control and anti‐CD3/CD28 (Clone HIT3 a/Clone CD28.2, ThermoFisher) stimulated condition as a positive control were taken along. Anti‐CD40 antibody (final concentration 1 µg/mL, Clone HB14, Miltenyi Biotec) was added 15 min before stimulation. PBMCs were stained with a panel consisting of 18 fluorescent conjugated antibodies and one viability dye (Supporting Information 1: Table S2) for 30 min at room temperature in the dark. Next, PBMCs were washed and fixated (Foxp3 staining buffer set, eBioscience) for 30 min at 4°C. Subsequently, PBMCs were washed and acquired on a FACSymphony (BD). Rainbow beads (BD) were used as reference to correct voltage settings during each acquisition. FlowAI R‐plugin was used for data quality control, which included anomaly detection via flow rate check (timestep 1/10 s), signal acquisition (threshold: 1300), and dynamic range (upper and lower limit). Data were manually analyzed using FlowJo v10.10.0 software. The gating of the dual activation marker combinations in Supporting Information 2: Figure S1B, Supporting Information 3: Figure S2A, and Supporting Information 5: Figure S4 was based on low background frequencies of each dual marker combination in the unstimulated control to reduce false positive detection.

### 2.3. Data and Statistical Analysis

The antigen‐specific CD4 or CD8 T cell response is depicted as the percentage of CD4 or CD8 T cells which are double positive for two markers (dual AIM combinations, Supporting Information 2: Figure S1, Supporting Information 3: Figure S2, and Supporting Information 5: Figure S4). In case of the combined dual AIMs (AIM^total^ and AIM^final^), OR Boolean gating function in FlowJo v10.10.0 software was used to combine T cells detected by certain dual AIM combinations ensuring that each cell was only counted once. Respective dual AIM combinations– or combined AIM–positive T cell percentages analyzed in the unstimulated condition were subtracted from antigen‐induced percentages. Each symbol (circles for the SARS‐CoV‐2 and CMV–infected cohort and squares for the SARS‐CoV‐2–vaccinated cohort) represents an individual sample. Lines connect data from the same individual. Pairwise multiple comparisons were performed using the Friedman test with Dunn’s multiple comparison correction. Comparisons in T cell responses detected for the same individuals were analyzed using the Wilcoxon matched‐pairs signed‐rank test, while tests comparing the two cohorts were performed using Mann–Whitney test. The Wilcoxon, Mann–Whitney, and Friedman tests were performed using GraphPad Prism software v10.3.1.

## 3. Results

### 3.1. CD40L Is Redundant for Detection of Antigen‐Specific CD4 T Cells in Combined AIM Analysis

In AIM assays, a combination of two activation markers is generally used to identify antigen‐specific T cells [1]. To determine whether combining multiple markers can capture a larger and more representative proportion of the antigen‐specific T cell response, a panel including six previously described activation markers (CD137, CD69, OX40, CD40L, CD25, and PD‐L1) was designed. This enabled the formation of 15 different dual marker combinations (Figure 1A). The AIM assay was performed on a heterogenous population of human PBMC samples, including individuals previously infected with both SARS‐CoV‐2 and CMV, as well as SARS‐CoV‐2–vaccinated individuals reflecting the sample material standardly used in AIM assays. PBMCs of the infected individuals were stimulated with either spike or CMV pp65 peptide pool (Supporting Information 2: Figure S1). For the vaccinated individuals, PBMC samples were collected 7 days after the second SARS‐CoV‐2 vaccination and stimulated with a spike peptide pool (Supporting Information 2: Figure S1 and Supporting Information 3: Figure S2A with dot plots of dual marker combinations). OR Boolean gating was used to combine the 15 dual marker combinations into a total AIM population (AIM^total^), ensuring each cell was only counted once (Figure 1A). First, the contribution of each of the six individual activation markers to the detection of antigen‐specific CD4 T cells was investigated by sequentially removing one marker from AIM^total^, resulting in the integration of 10 dual combinations (Figure 1B, C). Frequencies of spike– or CMV–specific CD4 T cells in infected individuals were significantly reduced when OX40, CD25, or PD‐L1 was removed, suggesting these markers contribute to CD4 T cell detection (Figure 1B). Excluding CD137, CD69, or CD40L did not significantly affect CD4 AIM^total^. In vaccinated individuals, removal of CD137, CD69, OX40, and CD25 resulted in a significantly lower spike‐specific CD4 T cell frequency (Figure 1C).

Figure 1CD40L has no additive value in combined AIM for detection of antigen‐specific CD4 T cells. (A) Graphical depiction of gating the 15 dual marker combinations of CD69, CD137, OX40, CD40L, and PD‐L1 and their integration to AIM^total^ by OR Boolean gating to detect antigen‐specific T cells. (B, C) Frequency of total AIM + CD4 T cells (AIM^total^) and sequential removal of one activation marker from AIM^total^ in infected individuals (*n* = 6) stimulated with either spike or CMV pp65 peptide pool for 24 h (B) and in individuals 7 days after the second SARS‐CoV‐2 vaccination (*n* = 6) stimulated with spike peptide pool for 18 h/24h (C). (D, E) Effect of removal of two activation markers from AIM^total^ on detection frequency in infected (D) and vaccinated (E) individuals. Unstimulated AIM frequencies were subtracted from stimulated AIM frequencies in all graphs. Friedman’s test was performed with Dunn’s multiple comparison correction. ns (nonsignificant, *p* > 0.05),  ^∗^(*p* ≤ 0.05),  ^∗∗^(*p* ≤ 0.01), and  ^∗∗∗^(*p* ≤ 0.001).

**Figure:**
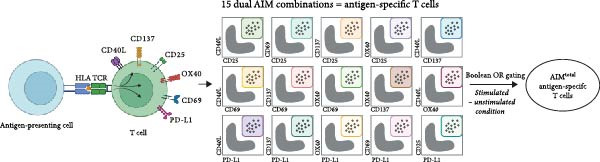
(A)

**Figure:**
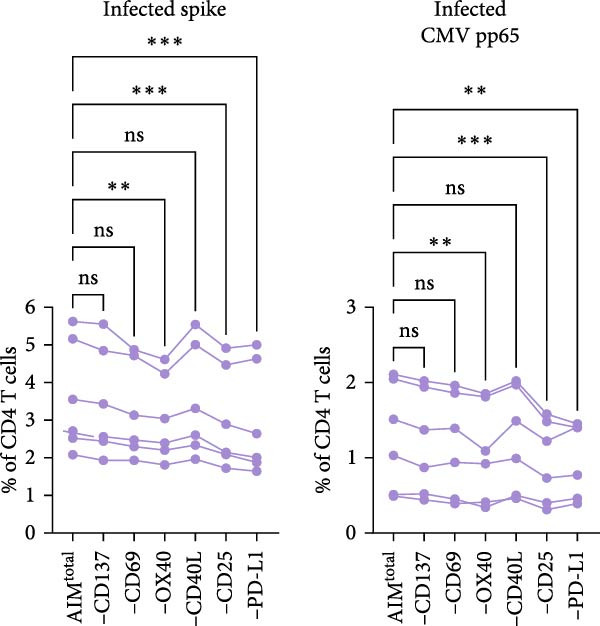
(B)

**Figure:**
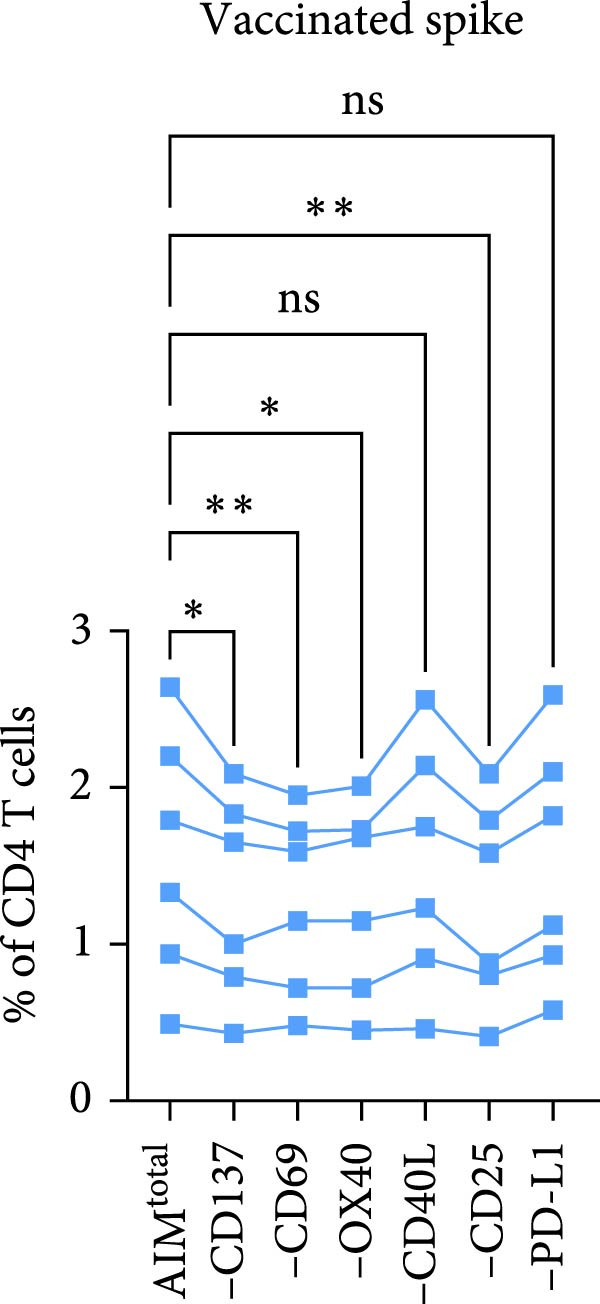
(C)

**Figure:**
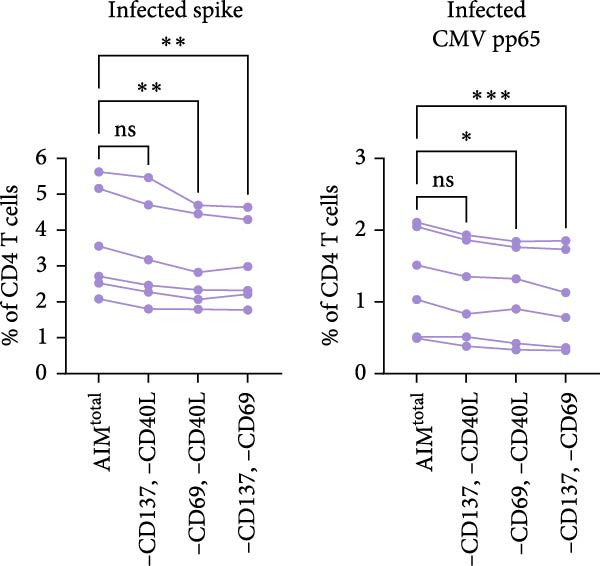
(D)

**Figure:**
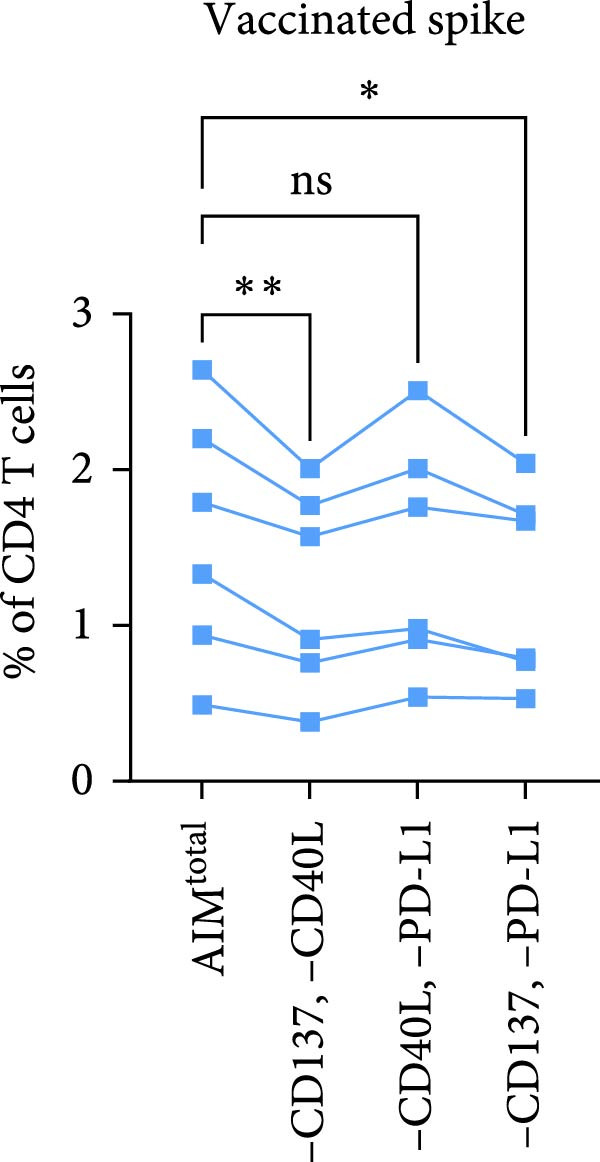
(E)

Next, the redundancy of certain activation markers was investigated using the markers that did not contribute to total spike– or CMV–specific CD4 T cell frequency in the initial analysis (Figure 1D, E). Removal of CD137 plus CD40L from AIM^total^ did not significantly affect the spike– or CMV–specific CD4 T cell frequencies in infected individuals, whereas CD40L plus PD‐L1 was redundant in detecting spike–specific CD4 T cells following SARS‐CoV‐2 vaccination (Figure 1D, E). To conclude, as only CD40L was redundant in both infected and vaccinated donors, the combination of OX40, CD69, CD25, CD137, and PD‐L1 is preferred for the detection of antigen‐specific CD4 T cells following different types of antigen exposure. Despite the relatively high background expression of CD69 (Supporting Information 3: Figure S2B,C), CD69 still added to the detection frequency of antigen‐specific CD4 T cells after the background signal was subtracted (Figure 1C, E). Therefore, it could be considered to exclude CD69 if increased sensitivity instead of higher detection frequency is desired.

### 3.2. PD‐L1 Contributes to CD4 T Cell Detection Frequency in SARS‐CoV‐2– and CMV–Infected Individuals

Since CD40L did not contribute to the detection frequency of antigen‐specific CD4 T cells following SARS‐CoV‐2 and CMV infection or SARS‐CoV‐2 vaccination, CD40L was excluded from AIM^total^. We proceeded with a combined AIM named AIM^final^, which included CD137, CD25, CD69, OX40, and PD‐L1. AIM^final^ detected a higher frequency of spike– or CMV pp65–specific CD4 T cells in infected individuals compared to each of the 15 dual marker combinations (Figure 2A, Supporting Information 4: Figure S3A). Among all dual marker combinations, CD69+/OX40+, PD‐L1+/CD25+, and PD‐L1+/OX40+ detected the highest percentage of antigen‐specific CD4 T cells but did not reach the level of AIM^final^. Similar to the results in infected individuals, AIM^final^ outperformed the dual marker combinations in spike–specific CD4 T cell frequency following SARS‐CoV‐2 vaccination (Figure 2B, Supporting Information 4: Figure S3A). Interestingly, dual marker combinations including PD‐L1 hardly detected any spike–specific CD4 T cells in vaccinated individuals. This was in contrast to infected individuals or SARS‐CoV‐2–vaccinated individuals who had also experienced COVID‐19 (Supporting Information 2: Figure S1B, Supporting Information 3: Figure S2A, and Supporting Information 4: Figure S3D,E), in whom dual marker combinations including PD‐L1 detected high spike–specific CD4 T cell frequencies (Figure 2A and Supporting Information 4: S3D, E).

Figure 2PD‐L1 enhances the detection frequency of antigen‐specific CD4 T cells in combined AIM in SARS‐CoV‐2– and CMV–infected but not SARS‐CoV‐2–vaccinated individuals. (A, B) Frequency of AIM + CD4 T cells using AIM^final^ (AIM^total^ minus CD40L) or dual marker combinations in SARS‐CoV‐2– and CMV–infected (A, *n* = 6) and SARS‐CoV‐2–vaccinated (B, *n* = 6) individuals stimulated with either spike or CMV pp65 peptide pool for 18/24 h. (C) Comparison of AIM^final^ with and without PD‐L1 between 7 days (T7d) and 6 months (T6m) after the second SARS‐CoV‐2 vaccination (*n* = 4). (D) Comparison of AIM^final^ with and without PD‐L1 between median 55 days (T55d) and 7 months (T7m) after SARS‐CoV‐2 infection in convalescent individuals. The Wilcoxon matched‐pairs signed‐rank test was performed. Unstimulated AIM frequencies were subtracted from stimulated AIM frequencies in all graphs. ns (nonsignificant, *p* > 0.05) and  ^∗^(*p* ≤ 0.05).

**Figure:**
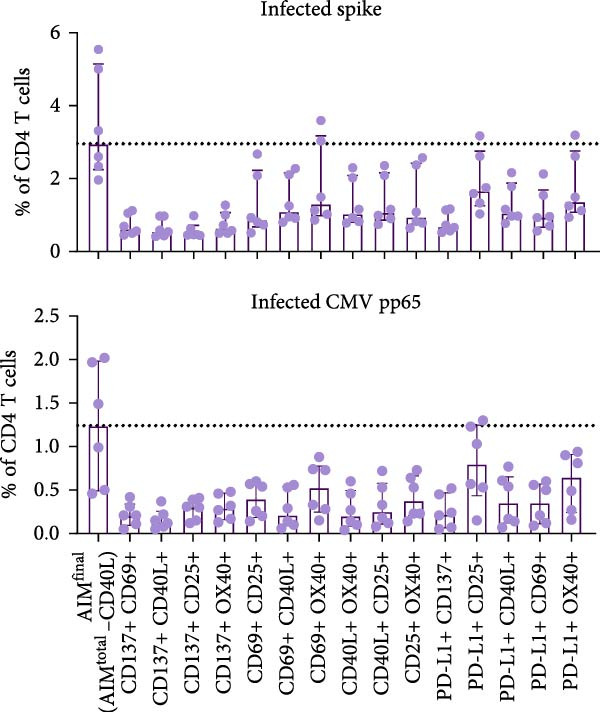
(A)

**Figure:**
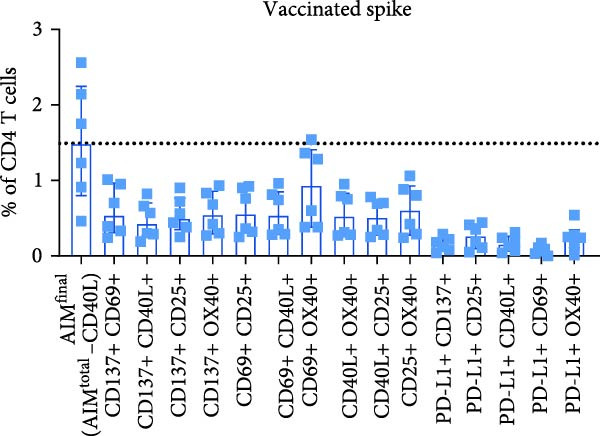
(B)

**Figure:**
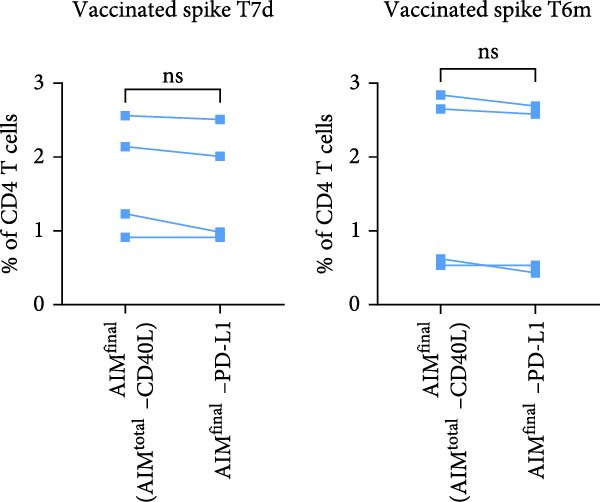
(C)

**Figure:**
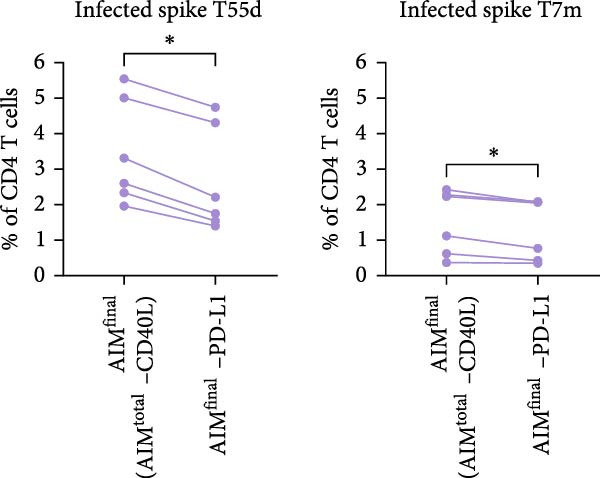
(D)

Next, we explored whether the difference in PD‐L1 expression on activated CD4 T cells between infected and vaccinated individuals could be related to the time since antigen exposure. In addition to samples early after vaccination (7 days), samples collected at a later timepoint (6 months) following the second vaccination were analyzed for PD‐L1 expression. Similar to day 7, PD‐L1 did not have a significant impact on spike–specific CD4 T cell detection 6 months after antigen exposure through mRNA vaccination (Figure 2C and Supporting Information 4: Figure S3F). Samples from SARS‐CoV‐2–infected individuals were also analyzed at an additional timepoint, around 7 months after infection. Similar to 55 days after infection, PD‐L1 significantly contributed to the detection of spike–specific CD4 T cells 7 months after SARS‐CoV‐2 infection (Figure 2D and Supporting Information 4: Figure S3G).

Besides the individual frequencies of each dual marker combination, their contribution to AIM^final^ was analyzed. Each dual marker combination was sequentially removed from AIM^final^, in both stimulated (with background subtraction) and unstimulated conditions. The combination PD‐L1+/CD25+ contributed highly to antigen‐specific CD4 T cell detection, since removal of this dual combination from AIM^final^ resulted in the most significantly reduced frequency of both spike– and CMV pp65–specific CD4 T cells after infection (Supporting Information 4: Figure S3B). In vaccinated individuals, the dual combination CD137+/CD25+ significantly contributed to detection of spike–specific CD4 T cells (Supporting Information 4: Figure S3C). In summary, PD‐L1 contributes to antigen‐specific CD4 T cell detection in SARS‐CoV‐2– or CMV–infected but not in SARS‐CoV‐2 mRNA–vaccinated individuals.

### 3.3. Dual Combination CD69 Plus CD25 Detects Majority of Antigen‐Specific CD8 T Cells

Detection of antigen‐specific CD8 T cells using AIM assays is often challenging due to high background expression of activation markers [21]. The most commonly used dual marker combination for antigen‐specific CD8 T cell detection is CD137+/CD69+. Taking background expression of activation markers into account, we investigated which combination of markers detected the highest frequency of antigen‐specific CD8 T cells. First, AIM^total^ CD8 T cell frequency was compared to each individual dual AIM combination in SARS‐CoV‐2– or CMV–infected and SARS‐CoV‐2–vaccinated individuals (Figure 3A and Supporting Information 5: Figure S4). Six out of 15 dual combinations, including CD137+/CD69+, CD137+/CD25+, CD69+/CD25+, PD‐L1+/CD137+, PD‐L1+/CD25+, and PD‐L1+/CD69+, detected spike– and CMV pp65–specific CD8 T cells (Figure 3A). The marker combination CD137+/CD69+ displayed high background expression in the unstimulated condition (Figure 3A and Supporting Information 5: Figure S4). Removal of CD137+/CD69+ from AIM^total^ resulted in significant increase in detection frequency of CMV–specific CD8 T cells (Figure 3B). Therefore, the dual combination CD137+/CD69+ was hereafter removed from AIM^total^, while other dual combinations with either CD137 or CD69 remained included. In addition, OX40 and CD40L were removed from AIM^total^, as they did not contribute to the detection of antigen‐specific CD8 T cells (Figure 3A, B).

Figure 3Dual combination of CD69+/CD25+ is sufficient for antigen‐specific CD8 T cell detection. (A) Frequency of AIM + CD8 T cells using AIM^total^ or dual marker combinations in SARS‐CoV‐2– and CMV–infected (*n* = 6) and SARS‐CoV‐2–vaccinated individuals (*n* = 6) either unstimulated or stimulated with spike or CMV pp65 peptide pool for 18/24 h. Median with interquartile range is shown. (B) Comparison between AIM^total^, AIM^total^ excluding the CD137+/CD69+ combination and AIM^total^ minus CD40L, and OX40 excluding CD137+/CD69+ in infected and vaccinated individuals. (C) Frequency of AIM^total^ minus CD40L, and OX40 excluding CD137+/CD69+ compared to removal of one marker in infected and vaccinated individuals. (D) Frequency of AIM + CD8 T cells using AIM^final^ (AIM^total^ minus CD40L, OX40, and CD137) or dual marker combinations in infected and vaccinated individuals. Median with interquartile range is shown. (E) Frequencies of AIM^total^, AIM^final^, and the dual combination CD69+/CD25+ in infected and vaccinated individuals either unstimulated or stimulated with peptide pool. The Wilcoxon matched‐pairs signed‐rank test was performed. Friedman’s test was performed with Dunn’s multiple comparison correction and unstimulated AIM frequencies were subtracted from stimulated AIM frequencies in B–D. ns (nonsignificant, *p* > 0.05),  ^∗^(*p* ≤ 0.05),  ^∗∗^(*p* ≤ 0.01), and  ^∗∗∗^(*p* ≤ 0.001).

**Figure:**
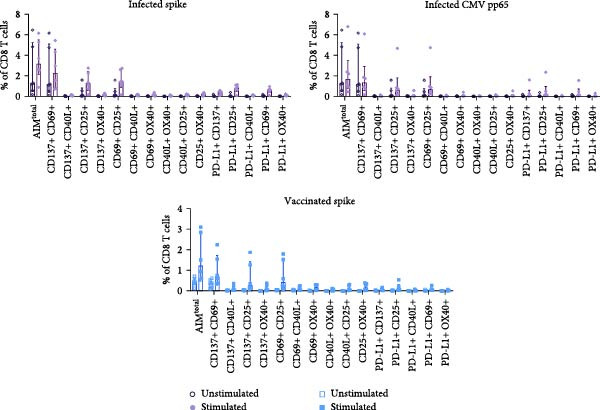
(A)

**Figure:**
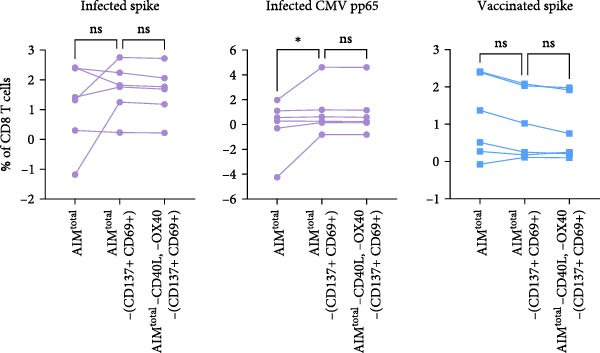
(B)

**Figure:**
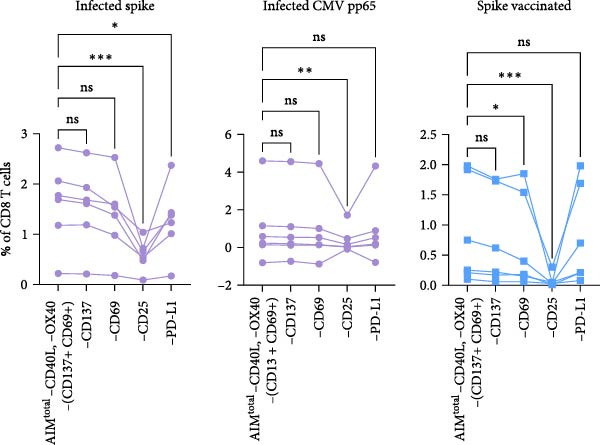
(C)

**Figure:**
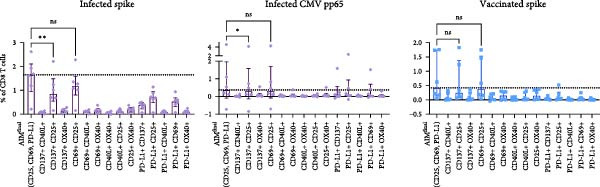
(D)

**Figure:**
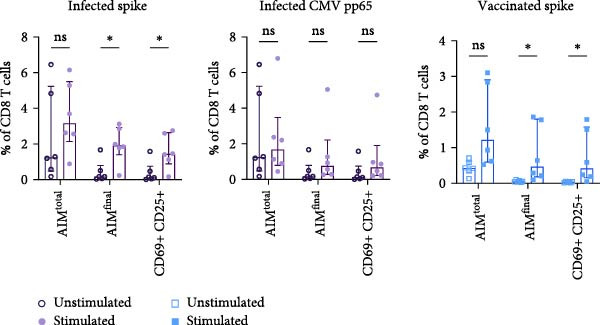
(E)

Next, the redundancy of the remaining activation markers: CD137, CD69, CD25, and PD‐L1, was tested. Removing CD25 significantly reduced the detection frequency of spike– and CMV–specific CD8 T cells in both infected and vaccinated individuals (Figure 3C). PD‐L1 contributed to the detection of spike‐specific CD8 T cells in infected individuals, and the removal of CD69 decreased the detection of spike–specific CD8 T cells in vaccinated individuals (Figure 3C). Since CD137 did not contribute to antigen‐specific CD8 T cell detection, it was excluded from AIM^total^, resulting in AIM^final^ for CD8 T cells which comprised CD69, CD25, and PD‐L1. AIM^final^ detected higher frequencies of spike– and CMV–specific CD8 T cells in infected individuals compared to the dual combination CD137+/CD25+ but reached similar frequencies compared to CD69+/CD25+ in both infected and vaccinated individuals (Figure 3D). In contrast to AIM^total^, AIM^final^ and the dual combination CD69+/CD25+ had lower background expression (Figure 3F). In summary, the dual combination CD137+/CD69+ should be avoided due to high background expression affecting antigen‐specific analysis. The majority of antigen‐specific CD8 T cells are detected by the dual combination CD25+/CD69+. However, since PD‐L1 also contributed to the detection frequency of spike–specific CD8 T cells in infected individuals, we suggest to include PD‐L1 for the comprehensive analysis of antigen‐specific CD8 T cells.

### 3.4. Certain Dual Activation Marker Combinations Have a Bias Toward a Specific T Helper Subset

Besides the variation in T cell frequency detected by different dual marker combinations, the influence of different dual marker combinations on detection of specific T helper phenotypes was investigated. Different T helper subsets were characterized using chemokine markers [22–25]: circulating T follicular helper (cTfh; CXCR5+), T helper 1 (Th1; CXCR5‐, CXCR3+, CCR6‐), T helper 1 like (Th1like; CXCR5‐, CXCR3+, CCR6+), T helper 2 (Th2; CXCR5‐, CCR4+, CXCR3‐, CCR6‐), T helper 9 (Th9; CXCR5‐, CCR4‐, CXCR3‐, CCR6+), and T helper 17 cells (Th17 cells; CXCR5‐, CCR4+, CXCR3‐, CCR6+) (Figure 4A, Supporting Information 6: Figure S5A). The spike–specific T helper phenotype was comparable between SARS‐CoV‐2–infected and –vaccinated individuals (Figure 4B, Supporting Information 6: S5B). The only notable difference was a higher frequency of Th2 cells among spike–specific CD4 T cells in vaccinated individuals compared to infected donors (Figure 4B, Supporting Information 6: Figure S5B). To determine whether different dual marker combinations preferentially detected specific T helper subsets, the T helper phenotype of each dual combination was compared to the T helper phenotype of AIM^final^ (AIM^total^‐CD40L) (Figure 4C, Supporting Information 7: Figure S6). Most dual marker combinations displayed similar cTfh, Th9, and Th17 frequencies compared to AIM^final^, indicating that most marker combinations detect similar cTfh, Th9, or Th17 frequencies. On the other hand, the dual combination CD137+/CD40L+ detected increased Tfh frequencies, while CD137+/CD25+ detected less Th9 cells and CD137+/CD69+ less Th17 cells compared to other marker combinations. Notably, Th2 cells were underrepresented in all dual combinations comprising PD‐L1 or CD40L. Marker combinations including OX40 were biased towards a Th1like phenotype. Lastly, the dual combinations CD137+/CD69+, CD137+/CD40L+, and CD69+/CD25+ detected a higher frequency of Th1 cells compared to AIM^final^ (Figure 4C). In conclusion, some dual marker combinations preferentially detected specific T helper phenotypes, creating a bias in the antigen‐specific CD4 T cell repertoire towards these T helper subsets. Using a combination of more than two AIMs may therefore phenotypically better represent the overall antigen‐specific CD4 T cell response.

Figure 4Dual marker combinations exhibit bias towards certain CD4 T helper phenotypes. (A) Representative plot of the gating strategy of AIM^+^ T helper populations. (B) Distribution of T helper populations within AIM^final^ (AIM^total^ minus CD40L) and all dual marker combinations in SARS‐CoV‐2–infected (*n* = 6) and –vaccinated (*n* = 6) individuals stimulated with spike peptide pool for 18/24 h. Median is shown. (C) Frequency of T helper subsets within AIM^final^ and dual marker combinations. SARS‐CoV‐2–infected and –vaccinated individuals were combined (*n* = 12). Median with interquartile range is shown. Frequency of each dual marker combination was compared to that of AIM^final^. Friedman’s test was performed with Dunn’s multiple comparison correction. ns (nonsignificant, *p* > 0.05),  ^∗^(*p* ≤ 0.05),  ^∗∗^(*p* ≤ 0.01),  ^∗∗∗^(*p* ≤ 0.001), and  ^∗∗∗∗^(*p* ≤ 0.0001).

**Figure:**
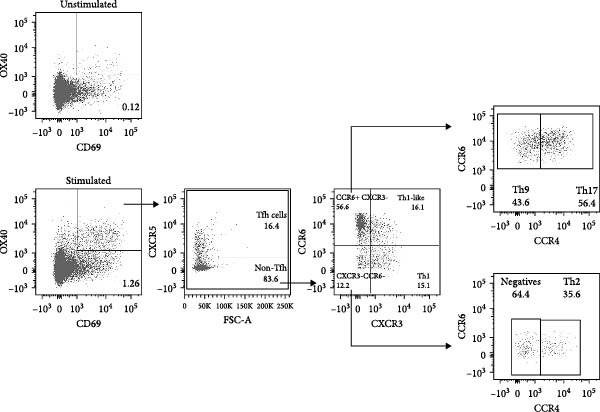
(A)

**Figure:**
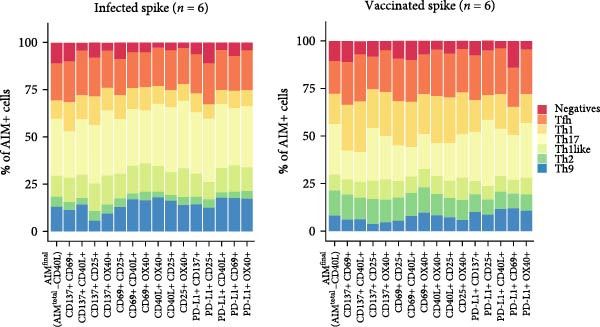
(B)

**Figure:**
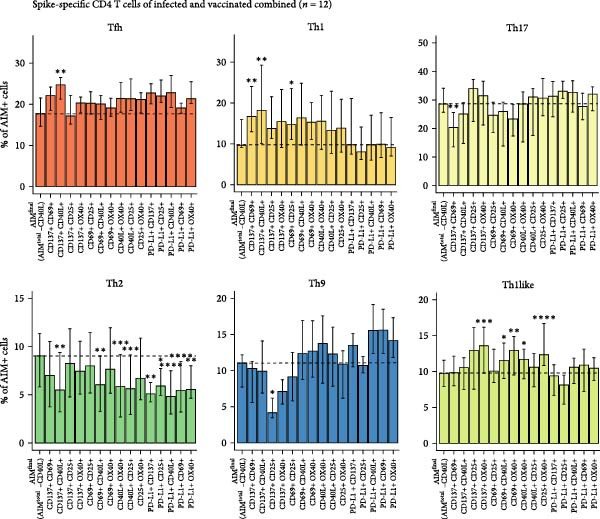
(C)

## 4. Discussion

In this study, we demonstrated that combining multiple AIMs increases the detection frequency of antigen‐specific T cells compared to single dual marker combinations. The expression of CD40L, CD137, OX40, CD25, CD69, and PD‐L1 was studied, revealing that inclusion of CD137, CD25, OX40, CD69, and PD‐L1 provides a comprehensive AIM panel for detecting both antigen‐specific CD4 and CD8 T cells following either natural infection or vaccination. Furthermore, we demonstrated that dual AIM combinations may favor detection of specific CD4 T helper subsets and underestimate the presence of others.

Following TCR stimulation, T cells upregulate activation markers that act as costimulatory receptors to proceed with T cell activation, promote proliferation, and drive T helper cell differentiation. Different T cell subsets preferably upregulate certain activation markers upon antigen stimulation [14, 26]. Therefore, the use of a limited number of activation markers reduces the likelihood of detecting the entire antigen‐specific T cell response. This could be circumvented by combining more than two activation markers. In addition, current limitations to the AIM assay such as skewing towards the detection of high affinity antigen‐specific CD4 T cells, bystander activation (activation of nonspecific T cells), and limited detection of very low‐frequency antigen‐specific CD4 T cells may also be ameliorated by combining multiple activation markers [27]. This needs, however, further investigation.

Although CD40L is often used as an activation marker for detecting antigen‐specific CD4 T cells, it is redundant when combined with other markers, in accordance with previous findings [26]. Excluding CD40L as activation marker from the assay has the advantage that cells do not have to be incubated with a CD40–blocking antibody to prevent CD40L internalization, thus simplifying the AIM assay procedure [28]. Moreover, CD137 did not add to the detection frequency of antigen‐specific CD4 T cells in SARS‐CoV‐2– or CMV–infected individuals. However, CD137 did contribute to spike–specific CD4 T cell detection following SARS‐CoV‐2 vaccination. Therefore, adding CD137 to the AIM panel will facilitate broad antigen‐specific T cell detection.

We established that PD‐L1 contributes to the detection frequency of antigen‐specific T cells. Interestingly, PD‐L1 only contributed in the context of natural infection and not following mRNA vaccination at an early and late time point after antigen exposure. PD‐L1 expression on CD4 and CD8 T cells has been previously associated with severe COVID‐19 disease [29]. However, the difference in PD‐L1 expression on T cells between infected and vaccinated donors remains unclear but is possibly caused by the nature of antigen exposure, namely, natural infection versus (mRNA) vaccination [3]. PD‐L1 expression is primarily known for its role in tumor survival, as upregulation of PD‐L1 on tumor cells attenuates the tumor‐specific T cell response. Expression of PD‐L1 on CD4 T cells restrains T cell proliferation and promotes Th17 differentiation at the expense of a Th1 phenotype [30, 31]. PD‐L1 has been scarcely used in AIM assays until now [4, 14, 15]. Our data suggest that PD‐L1 is less expressed by Th2 cells. Additionally, we observed trends indicating that PD‐L1 is preferentially expressed by Th17, Th9, and cTfh T cells, while it is less expressed by Th1 and Th1–like cells, which aligns with previous research [4, 31]. Moreover, PD‐L1 and CD25 coexpression identified antigen‐specific CD4 T cells not detected by other dual marker combinations.

PD‐L1 expression also contributed to the detection of spike–specific CD8 T cells in SARS‐CoV‐2 infected individuals, a finding to our knowledge not previously demonstrated. Specifically, PD‐L1 in combination with CD69 or CD25 detected antigen‐specific CD8 T cells following SARS‐CoV‐2 infection. Notably, the CD137+/CD69+ dual combination had a very high background signal, making it challenging to detect low‐frequency antigen‐specific CD8 T cell responses. Moreover, it has been described that CD137 in combination with CD25, rather than CD69, correlates better with IFN‐*γ*–expressing T cells in ELISpot and with intracellular TNF*α* expression [12]. Although CD137 in combination with CD69 is often used for antigen‐specific CD8 T cell detection [12], we propose to avoid this specific combination in analysis of antigen‐specific CD8 T cells. We demonstrated that the dual combination of CD69 plus CD25 is sufficient for antigen‐specific CD8 T cell detection. However, PD‐L1 contributed to the detection of spike–specific CD8 T cells in infected individuals. Addition of PD‐L1 could, therefore, broaden the spectrum of antigen‐specific CD8 T cells detected.

Lastly, use of dual marker combinations, or a limited selection of markers, may underestimate or overestimate certain CD4 T helper subsets in the antigen‐specific CD4 T cell analysis. For example, CD40L+/CD137+ detected more cTfh and Th1 cells while detecting fewer Th2. The increased detection frequency of cTfh using CD40L might be explained by the high expression of CD40L on Tfh cells and its importance for Tfh–B cell interaction during germinal center (GC) responses [32]. The binding of CD40L to CD40 on B cells is important for driving GC B cell responses [33]. Indeed, Lemieux and colleagues [26] also detected increased CD40L expression on cTfh cells [26]. Additionally, CD69 and CD137 coexpression detected more Th1 cells at the expense of Th17 cells. Studies using CD69 and CD40L detected mainly spike–specific Th1 cells [1, 34, 35]. Consistently, CD40L has been shown to be primarily expressed on IFN‐*γ*– and TNF–expressing Th1 cells [36]. Thus, combining multiple dual marker combinations in the AIM assay may mitigate phenotypic detection bias. To note, T helper subsets were distinguished in this study by their differential chemokine expression [22–25]. Additionally, master transcription factors dictating the T helper differentiation could be measured to support the distinction of T helper subsets [37]. A higher proportion of Th2 cells was observed among spike–specific CD4 T cells in vaccinated individuals compared to those who had recovered from SARS‐CoV‐2 infection. This difference may be attributed to the distinct sampling time points: Vaccinated individuals were sampled 7 days after receiving their second dose, whereas infected individuals were sampled 55 days postinfection. It is possible that Th2 cells contract earlier than other T helper subsets following antigen exposure or migrate to tissue sites, leading to their reduced presence in the periphery at later time points. Alternatively, the nature of antigen exposure—vaccination versus natural infection—may influence T cell polarization. Supporting this, a higher frequency of central memory Th2 cells has previously been reported in vaccinated individuals compared to those who were naturally infected [38].

To conclude, combining multiple AIMs enhances the detection frequencies of CD4 T cells. While a dual marker combination may be proficient in detecting specific T cell subsets of interest, combining multiple activation markers provides a comprehensive tool for detecting antigen‐specific T cells and is applicable in various antigenic and pathogenic contexts.

## Ethics Statement

Study protocols were approved by the ethics advisory council of Sanquin Blood Supply Foundation and the medical ethical committee (NL74974.018.20 and EudraCT 2021‐001102‐30), respectively, for the infected and vaccinated study cohorts. All participants provided written informed consent.

## Disclosure

All authors listed have made a substantial, direct, and intellectual contribution to the work and approved it for publication.

## Conflicts of Interest

Filip Eftimov, Gertjan Wolbink, S. Marieke van Ham, and Taco W. Kuijpers report (governmental) grants from ZonMw to study immune response after SARS‐CoV‐2 vaccination in autoimmune diseases. Filip Eftimov also reports grants from Prinses Beatrix Spierfonds, CSL Behring, Kedrion, Terumo BCT, Grifols, Takeda Pharmaceutical Company, and GBS‐CIDP Foundation; consulting fees from UCB Pharma and CSl Behring; and honoraria from Grifols. All other authors report no conflicts of interest.

## Author Contributions

Christine Kreher, Laura Y. L. Kummer, and Anja ten Brinke designed the study. Christine Kreher, Laura Y. L. Kummer, Maryse Tempert, and Veronique A. L. Konijn performed the experiments. Christine Kreher and Laura Y. L. Kummer analyzed the data and prepared the figures. Gertjan Wolbink, Filip Eftimov, and Taco W. Kuijpers recruited donors and provided samples. Anja ten Brinke and S. Marieke van Ham supervised the study and acquired funding. Christine Kreher and Laura Y. L. Kummer wrote the original manuscript. Christine Kreher, Laura Y. L. Kummer, Theo Rispens, Taco W. Kuijpers, S. Marieke van Ham, and Anja ten Brinke revised and edited the manuscript. Christine Kreher and Laura Y. L. Kummer contributed equally to this work and should be listed as cofirst authors.

## Funding

The authors declare financial support was received for the research, authorship, and/or publication of this article. This research project was supported by ZonMw (The Netherlands Organization for Health Research and Development) Project Number #10430072010007. Anja ten Brinke and S. Marieke van Ham have received funding for Christine Kreher from the European Union’s Horizon 2020 research and innovation program under the Marie Skłodowska‐Curie Grant Agreement No. 860003.

## Supporting Information

Additional supporting information can be found online in the Supporting Information section.

## Supporting information


**Supporting Information 1** Table S1: Age and sex of individuals included in this study. Table S2: Antibodies used in the AIM assay for detection and characterization of antigen‐specific T cells.

**Supporting Information 2** Figure S1: Gatingof dual marker combinations in SARS‐CoV‐2– and CMV–infected individuals. (A) Representative gating of CD4 and CD8 T cells. (B) Representative gating of the 15 dual marker combinations from total CD4 T cells in unstimulated and spike or CMV pp65 peptide pool stimulated condition. Depiction was based on a selection of 100,000 cells.

**Supporting Information 3** Figure S2: Gating of dual marker combinations in SARS‐CoV‐2–vaccinated individuals and background of activation markers in combined AIM. (A) Representative gating of the 15 dual marker combinations from total CD4 T cells in unstimulated and spike peptide pool stimulated condition in SARS‐CoV‐2–vaccinated individual. Depiction was based on a selection of 100,000 cells. (B) Frequency of background (unstimulated) AIM+ CD4 T cells analyzed by AIM^total^ and sequential removal of one activation marker from AIM^total^ in infected (*n* = 6) and vaccinated (*n* = 6) individuals. (C) Stimulation index (frequency of spike or CMV pp65–stimulated/unstimulated CD4 T cells) analyzed by AIM^total^ and sequential removal of one activation marker from AIM^total^ in infected (*n* = 6) and vaccinated (*n* = 6) individuals. Friedman’s test was performed with Dunn’s multiple comparison correction. ns (nonsignificant, *p* >  0.05), ^∗^(*p* ≤ 0.05),  ^∗∗^(*p* ≤ 0.01),  ^∗∗∗^(*p* ≤ 0.001), and  ^∗∗∗∗^(*p* ≤ 0.0001).

**Supporting Information 4** Figure S3: Combined AIM for detection of antigen‐specific CD4 T cells in SARS‐CoV‐2– and CMV–infected and SARS‐CoV‐2–vaccinated individuals. (A) Frequency of AIM+ CD4 T cells using AIM^final^ (AIM^total^ minus CD40L) or dual marker combinations in SARS‐CoV‐2– and CMV–infected (*n* = 6) and SARS‐CoV‐2–vaccinated individuals (*n* = 6) either unstimulated or stimulated with spike or CMV pp65 peptide pool for 18/24 h. Median with interquartile range is shown. (B, C) Contribution of dual marker combination to AIM^final^ in SARS‐CoV‐2– and CMV–infected (B) or SARS‐CoV‐2–vaccinated (C) individuals. Frequency of remaining combined AIM after subtraction of each dual marker combination was compared to that of AIM^final^ for stimulated (background subtracted) and unstimulated condition. Median with interquartile range is shown. (D) Frequency of AIM+ CD4 T cells analyzed either using combined AIM^final^ or dual marker combinations of SARS‐CoV‐2–vaccinated individuals with previous SARS‐CoV‐2 infection (*n* = 2) stimulated with spike peptide pool for 18 h. Median is shown. (E) Comparison of AIM^final^ with and without PD‐L1 in SARS‐CoV‐2–vaccinated individuals with (*n* = 2) and without (*n* = 6) previous SARS‐CoV‐2 infection stimulated with spike peptide pool for 18/24 h. (F, G) Frequency of AIM+ CD4 T cells analyzed either using AIM^final^ or dual marker combinations of individuals 7 days (T7d) and 6 months (T6m) after second SARS‐CoV‐2 vaccination (F, *n* = 4) and of individuals 55 days (T55d) and 7 months (T7m) after SARS‐CoV‐2 infection (G, *n* = 6) stimulated with spike peptide pool for 24 h. Median with interquartile range is shown. Unstimulated AIM frequencies were subtracted from stimulated AIM frequencies in B–G. Friedman’s test was performed with Dunn’s multiple comparison correction in B and C. ns (nonsignificant, *p* >  0.05),  ^∗^(*p* ≤ 0.05),  ^∗∗^(*p* ≤ 0.01),  ^∗∗∗^(*p* ≤ 0.001), and  ^∗∗∗∗^(*p* ≤ 0.0001).

**Supporting Information 5** Figure S4: Gating of dual marker combinations in CD8 T cells of SARS‐CoV‐2– and CMV–infected individuals. Representative gating of the 15 dual marker combinations from total CD8 T cells in unstimulated and spike or CMV pp65 peptide pool stimulated condition. Depiction was based on a selection of 70,000 cells.

**Supporting Information 6** Figure S5: Fluorescence minus one (FMO) for the chemokines CXCR5, CXCR3, CCR6, and CCR4 used for distinguishing T helper subsets and comparison of CD4 T helper subsets between infected and vaccinated individuals. (A) Representative plots of FMOs on total CD4 T cells or AIM+ CD4 T cells (CD69+ OX40+) from a SARS‐CoV‐2–infected individual stimulated with spike peptide pool for 24 h based on gating strategy shown in Figure 4A. (B) Frequency of T helper subsets within AIM^final^ (AIM^total^ minus CD40L) in SARS‐CoV‐2–vaccinated (*n* = 6) and –infected (*n* = 6) individuals stimulated with spike peptide pool for 18/24 h. Median with interquartile range is shown. Mann–Whitney test was performed. ns (nonsignificant, *p* >  0.05) and  ^∗∗^(*p* ≤ 0.01).

**Supporting Information 7** Figure S6: Antigen‐specific CD4 T helper phenotype detected by dual marker combinations in SARS‐CoV‐2–infected and –vaccinated individuals. (A, B) Frequency of T helper subsets within AIM^final^ (AIM^total^ minus CD40L) and each dual marker combination, analyzed in SARS‐CoV‐2–infected (A, *n* = 6) and –vaccinated individuals (B, *n* = 6). Individual data points and median with interquartile range are shown.

## Data Availability

The data that support the findings of this study are available from the corresponding author upon reasonable request.
