# Supplementary material for: Combining Activation‐Induced Markers With PD‐L1 Selectively Enhances Detection of Antigen‐Specific T Cells in Virus‐Infected Individuals
Source: J Immunol Res. 2026 Feb 4;2026:1284907. doi: 10.1155/jimr/1284907 (PMC13140888; doi:10.1155/jimr/1284907)
Supplement: Supplementary file 1 — Supporting Information 1 Table S1: Age and sex of individuals included in this study. Table S2: Antibodies used in the AIM assay for detection and characterization of antigen‐specific T cells. [file JIMR-2026-1284907-s005.docx]

# **Supplementary tables**

**Table 1: Characteristics of individuals included in this study**

|  | COVID-19 convalescent individuals with previous CMV infection (n=6) | SARS-CoV-2 mRNA vaccinated individuals | |
| --- | --- | --- | --- |
|  |  | No previous COVID-19 infection (n=6) | Previous COVID-19 infection (n=2) |
| Age in years, mean (SD) | 57.4 (2.8) | 51.5 (4.8) | 51.5 (0.5) |
| Female sex, n (%) | 2 (33 %) | 3 (50 %) | 1 (50 %) |

**Table 2: Flow cytometry panel used in the AIM assay**

| **Antibody** | **Clone** | **Fluochrome** | **Dilution** | **Vendor** | **Catalog no** |
| --- | --- | --- | --- | --- | --- |
| CD4 | SK3 | BUV395 | 1:50 | BD | 563550 |
| CD8 | RPA-T8 | APC | 1:200 | BD | 555369 |
| CD27 | L128 | BUV563 | 1:400 | BD | 748705 |
| CD14 | M5E2 | AF700 | 1:200 | BD | 561029 |
| ICOS (CD278) | DX29 | BUV661 | 1:25 | BD | 741664 |
| CD69 | FN50 | BUV737 | 1:400 | BD | 612817 |
| CD3 | SK7 | BUV805 | 1:800 | BD | 612893 |
| CD40L (CD154) | 24-31 | BV421 | 1:200 | Biolegend | 310824 |
| CD25 | 2A3 | BV480 | 1:400 | BD | 746644 |
| Viability dye |  | LIVE/DEAD™ Fixable Near-IR | 1:1000 | Thermo Fisher | L34976 |
| CCR4 (CD194) | L291H4 | PE-Dazzle | 1:100 | Biolegend | 359420 |
| OX40 (CD134) | ACT35 | BUV615 | 1:100 | BD | 751455 |
| CXCR5 (CD185) | RF8B2 | BB515 | 1:50 | BD | 564625 |
| CD45RA | HI100 | BV786 | 1:400 | BD | 563870 |
| CCR6 (CD196) | 11A9 | BV605 | 1:50 | BD | 562724 |
| PD1 | EH12.1 | BB700 | 1:100 | BD | 566460 |
| PD-L1 (CD274) | 29E.2A3 | PE | 1:3200 | Biolegend | 329706 |
| CD137  (4-1BB) | 4B4-1 | BV650 | 1:200 | BD | 564092 |
| CXCR3 (CD183) | 1C6/CXCR3 | PE-Cy7 | 1:100 | BD | 560831 |
