# Supplementary figures and images for: Combining Activation‐Induced Markers With PD‐L1 Selectively Enhances Detection of Antigen‐Specific T Cells in Virus‐Infected Individuals
Source: J Immunol Res. 2026 Feb 4;2026:1284907. doi: 10.1155/jimr/1284907 (PMC13140888; doi:10.1155/jimr/1284907)

Figure S1

A

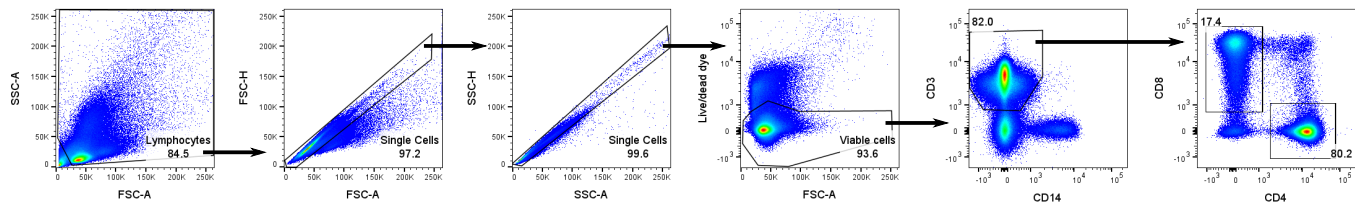

B

CD4 T cells of SARS-CoV-2 and CMV infected individual

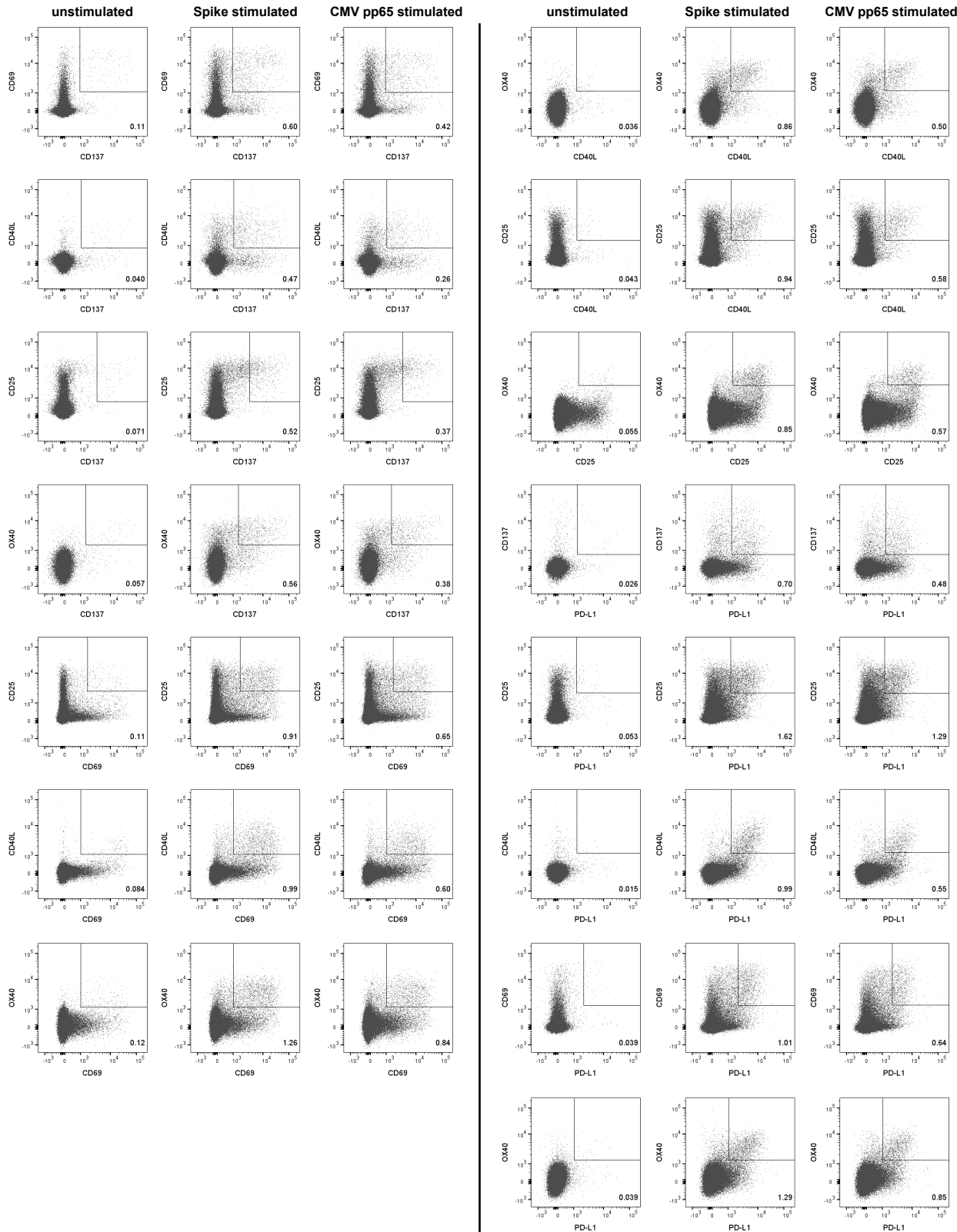

Supplement: Supplementary file 2 — Supporting Information 2 Figure S1: Gatingof dual marker combinations in SARS‐CoV‐2– and CMV–infected individuals. (A) Representative gating of CD4 and CD8 T cells. (B) Representative gating of the 15 dual marker combinations from total CD4 T cells in unstimulated and spike or CMV pp65 peptide pool stimulated condition. Depiction was based on a selection of 100,000 cells. [file JIMR-2026-1284907-s004.pdf]

# A

CD4 T cells of SARS-CoV-2 vaccinated individual

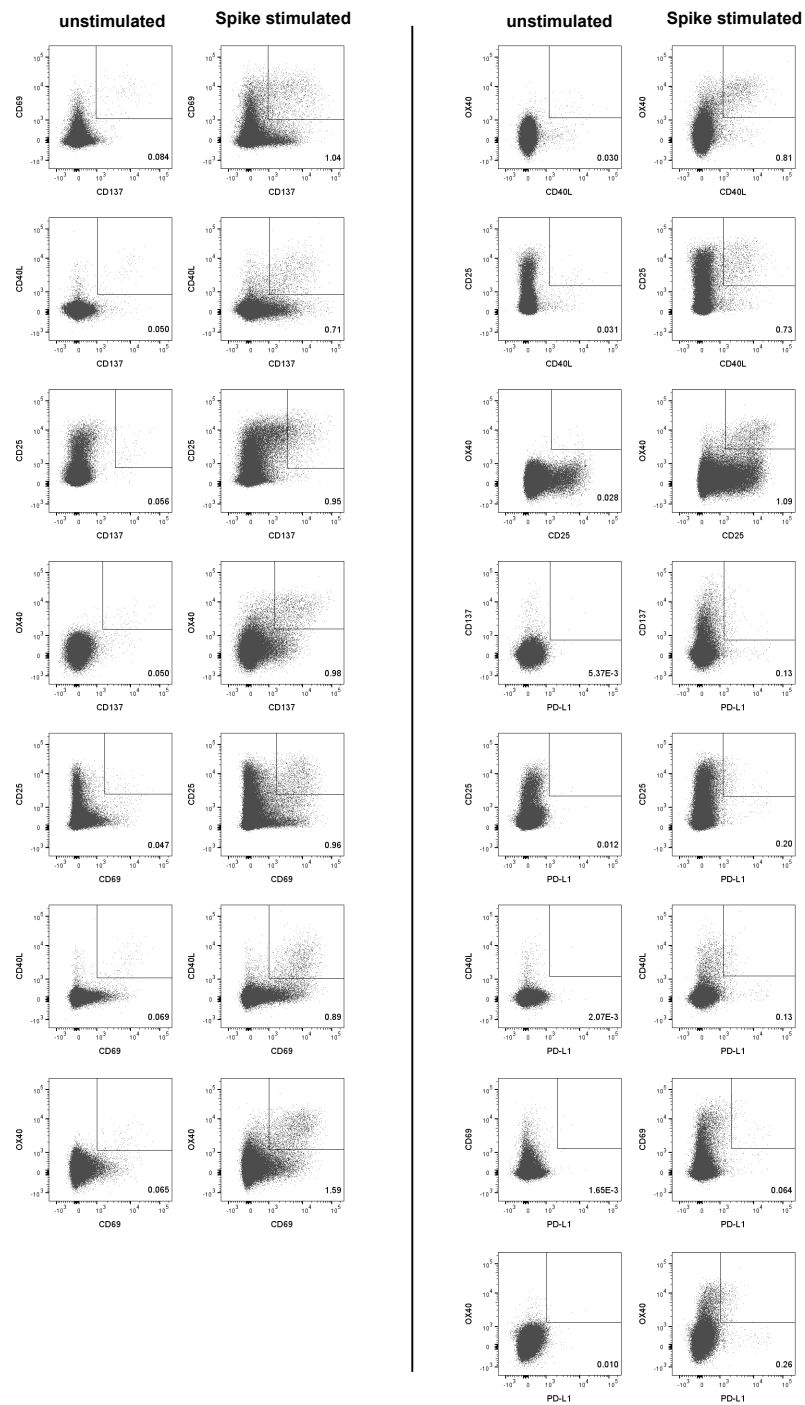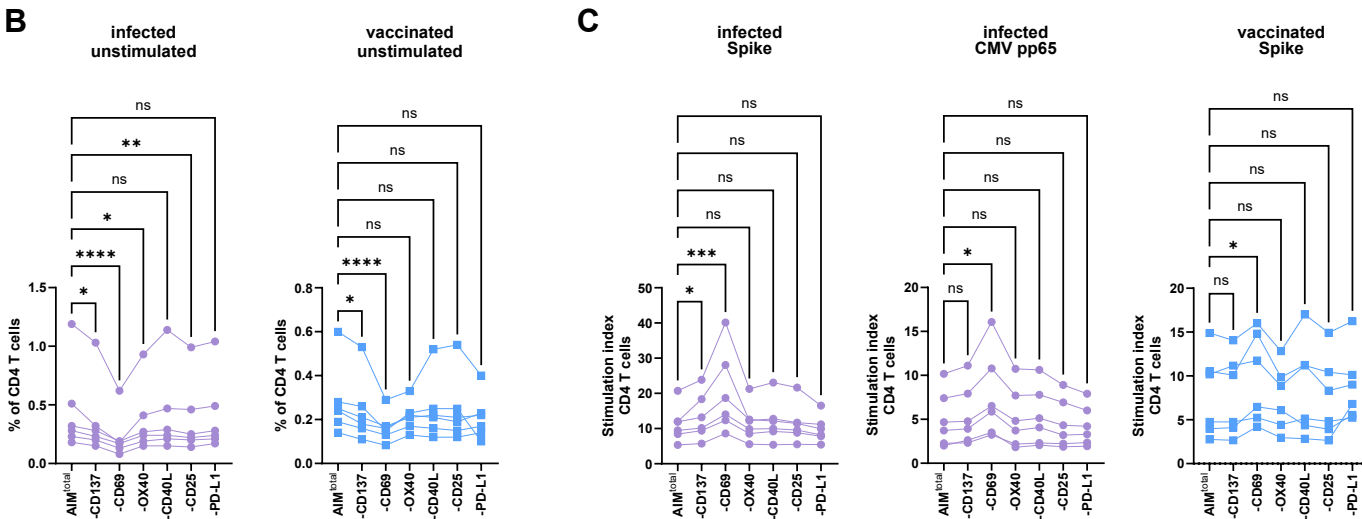

Supplement: Supplementary file 3 — Supporting Information 3 Figure S2: Gating of dual marker combinations in SARS‐CoV‐2–vaccinated individuals and background of activation markers in combined AIM. (A) Representative gating of the 15 dual marker combinations from total CD4 T cells in unstimulated and spike peptide pool stimulated condition in SARS‐CoV‐2–vaccinated individual. Depiction was based on a selection of 100,000 cells. (B) Frequency of background (unstimulated) AIM+ CD4 T cells analyzed by AIMtotal and sequential removal of one activation marker from AIMtotal in infected (n = 6) and vaccinated (n = 6) individuals. (C) Stimulation index (frequency of spike or CMV pp65–stimulated/unstimulated CD4 T cells) analyzed by AIMtotal and sequential removal of one activation marker from AIMtotal in infected (n = 6) and vaccinated (n = 6) individuals. Friedman’s test was performed with Dunn’s multiple comparison correction. ns (nonsignificant, p > 0.05), ∗(p ≤ 0.05), ∗∗(p ≤ 0.01), ∗∗∗(p ≤ 0.001), and ∗∗∗∗(p ≤ 0.0001). [file JIMR-2026-1284907-s003.pdf]

Figure S3

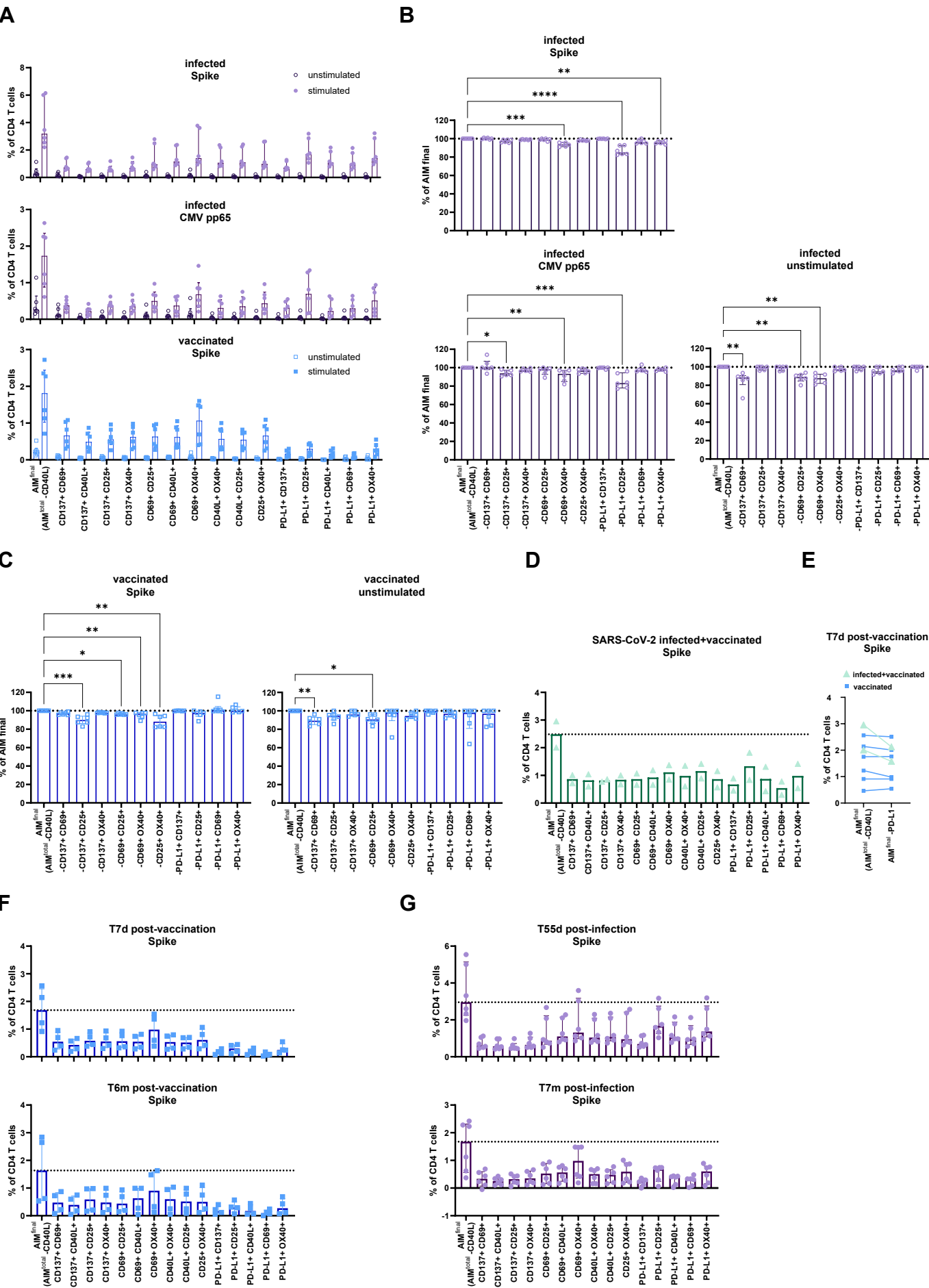

Supplement: Supplementary file 4 — Supporting Information 4 Figure S3: Combined AIM for detection of antigen‐specific CD4 T cells in SARS‐CoV‐2– and CMV–infected and SARS‐CoV‐2–vaccinated individuals. (A) Frequency of AIM+ CD4 T cells using AIMfinal (AIMtotal minus CD40L) or dual marker combinations in SARS‐CoV‐2– and CMV–infected (n = 6) and SARS‐CoV‐2–vaccinated individuals (n = 6) either unstimulated or stimulated with spike or CMV pp65 peptide pool for 18/24 h. Median with interquartile range is shown. (B, C) Contribution of dual marker combination to AIMfinal in SARS‐CoV‐2– and CMV–infected (B) or SARS‐CoV‐2–vaccinated (C) individuals. Frequency of remaining combined AIM after subtraction of each dual marker combination was compared to that of AIMfinal for stimulated (background subtracted) and unstimulated condition. Median with interquartile range is shown. (D) Frequency of AIM+ CD4 T cells analyzed either using combined AIMfinal or dual marker combinations of SARS‐CoV‐2–vaccinated individuals with previous SARS‐CoV‐2 infection (n = 2) stimulated with spike peptide pool for 18 h. Median is shown. (E) Comparison of AIMfinal with and without PD‐L1 in SARS‐CoV‐2–vaccinated individuals with (n = 2) and without (n = 6) previous SARS‐CoV‐2 infection stimulated with spike peptide pool for 18/24 h. (F, G) Frequency of AIM+ CD4 T cells analyzed either using AIMfinal or dual marker combinations of individuals 7 days (T7d) and 6 months (T6m) after second SARS‐CoV‐2 vaccination (F, n = 4) and of individuals 55 days (T55d) and 7 months (T7m) after SARS‐CoV‐2 infection (G, n = 6) stimulated with spike peptide pool for 24 h. Median with interquartile range is shown. Unstimulated AIM frequencies were subtracted from stimulated AIM frequencies in B–G. Friedman’s test was performed with Dunn’s multiple comparison correction in B and C. ns (nonsignificant, p > 0.05), ∗(p ≤ 0.05), ∗∗(p ≤ 0.01), ∗∗∗(p ≤ 0.001), and ∗∗∗∗(p ≤ 0.0001). [file JIMR-2026-1284907-s002.pdf]

Figure S4

CD8 T cells of SARS-CoV-2 and CMV infected individual

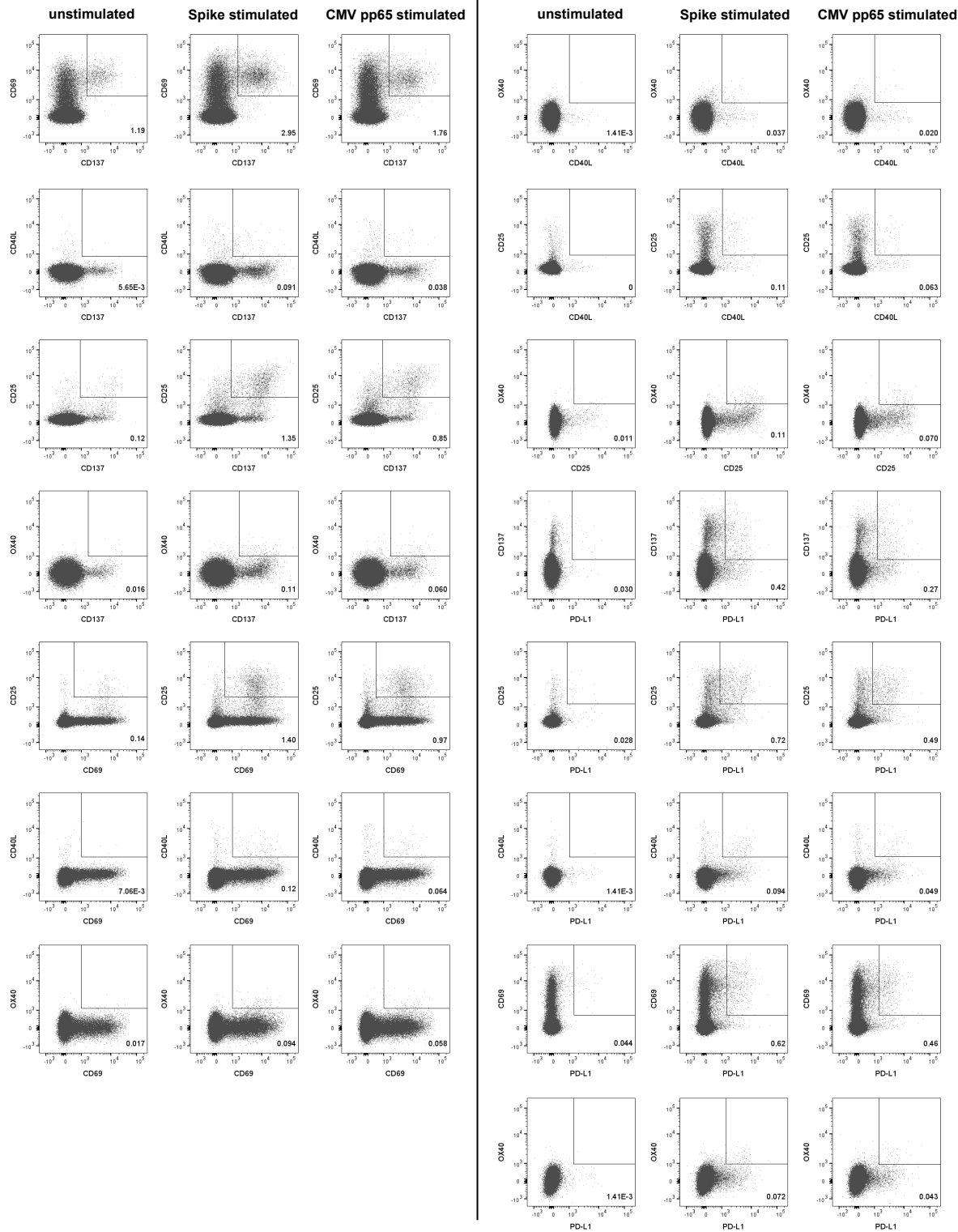

Supplement: Supplementary file 5 — Supporting Information 5 Figure S4: Gating of dual marker combinations in CD8 T cells of SARS‐CoV‐2– and CMV–infected individuals. Representative gating of the 15 dual marker combinations from total CD8 T cells in unstimulated and spike or CMV pp65 peptide pool stimulated condition. Depiction was based on a selection of 70,000 cells. [file JIMR-2026-1284907-s001.pdf]

Figure S5

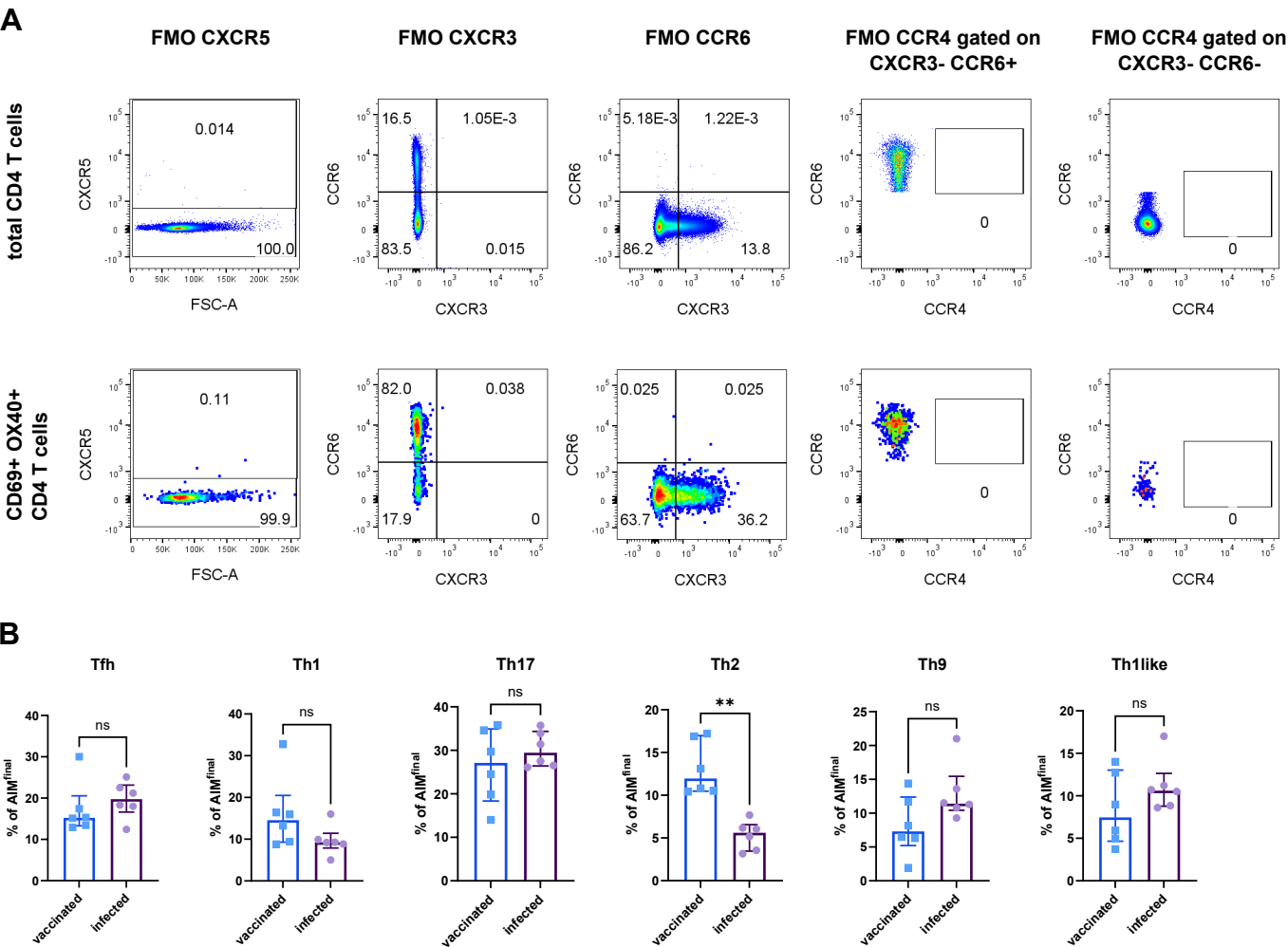

Supplement: Supplementary file 6 — Supporting Information 6 Figure S5: Fluorescence minus one (FMO) for the chemokines CXCR5, CXCR3, CCR6, and CCR4 used for distinguishing T helper subsets and comparison of CD4 T helper subsets between infected and vaccinated individuals. (A) Representative plots of FMOs on total CD4 T cells or AIM+ CD4 T cells (CD69+ OX40+) from a SARS‐CoV‐2–infected individual stimulated with spike peptide pool for 24 h based on gating strategy shown in Figure 4A. (B) Frequency of T helper subsets within AIMfinal (AIMtotal minus CD40L) in SARS‐CoV‐2–vaccinated (n = 6) and –infected (n = 6) individuals stimulated with spike peptide pool for 18/24 h. Median with interquartile range is shown. Mann–Whitney test was performed. ns (nonsignificant, p > 0.05) and ∗∗(p ≤ 0.01). [file JIMR-2026-1284907-s007.pdf]

Figure S6

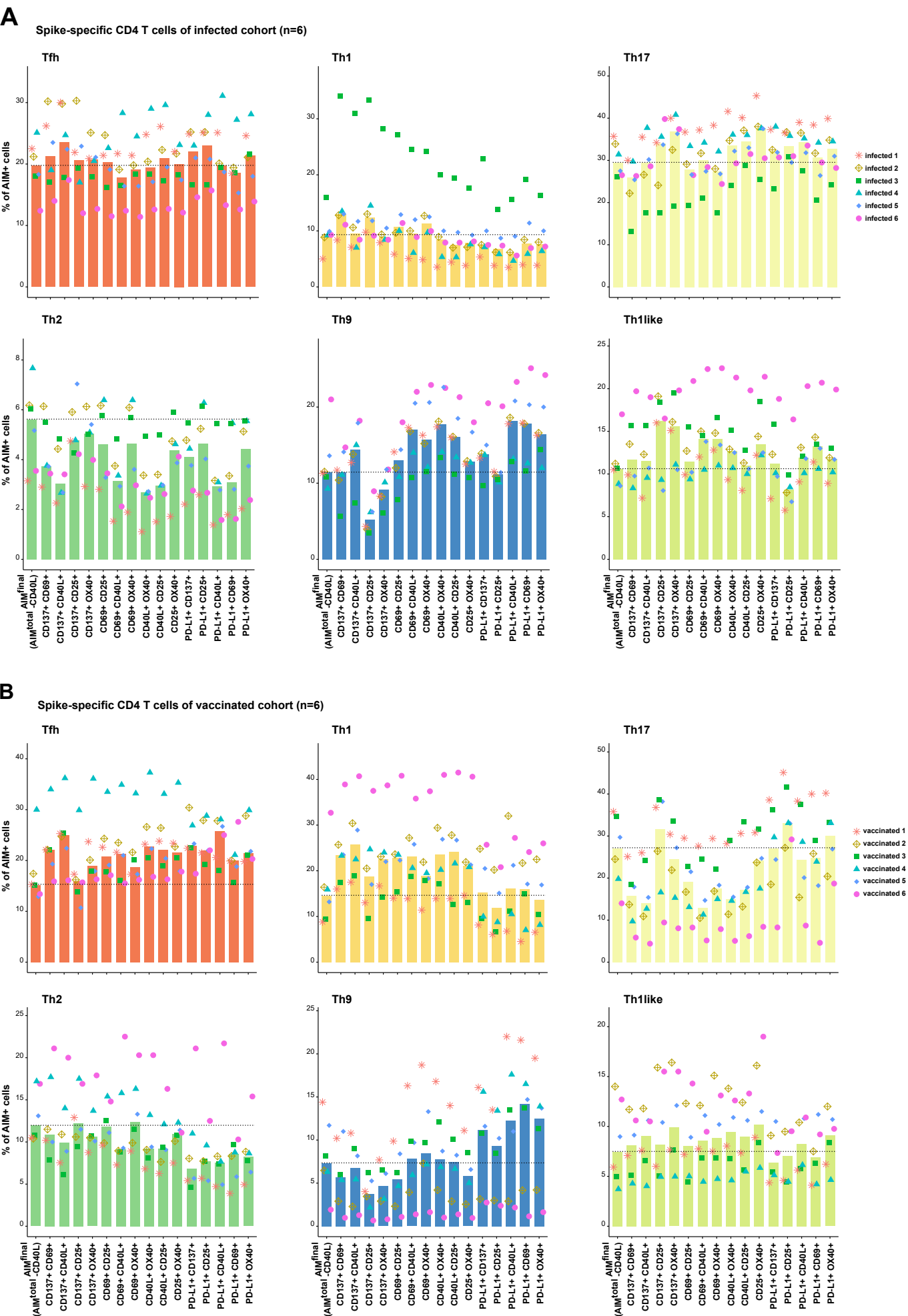

Supplement: Supplementary file 7 — Supporting Information 7 Figure S6: Antigen‐specific CD4 T helper phenotype detected by dual marker combinations in SARS‐CoV‐2–infected and –vaccinated individuals. (A, B) Frequency of T helper subsets within AIMfinal (AIMtotal minus CD40L) and each dual marker combination, analyzed in SARS‐CoV‐2–infected (A, n = 6) and –vaccinated individuals (B, n = 6). Individual data points and median with interquartile range are shown. [file JIMR-2026-1284907-s006.pdf]
